# Risk Stratification of Patients Being Considered for Open Abdominal Aortic Aneurysm Repair

**DOI:** 10.3390/jcm15166206

**Published:** 2026-08-11

**Authors:** Mitri K. Khoury, R. Trey Rogers, Hasan Aldailami, Tiziano Tallarita, Shiv Patel

**Affiliations:** 1 Division of Vascular and Endovascular Surgery, Arizona State University, Tempe, AZ 85287, USA; 2 HonorHealth Heart Care, Division of Vascular and Endovascular Surgery, 7351 E. Osborn Road Suite 100, Scottsdale, AZ 85251, USA

**Keywords:** abdominal aortic aneurysm, open repair, endovascular repair, fitness, risk stratification

## Abstract

**Background/Objectives**: Endovascular repair (EVAR) is the default for most abdominal aortic aneurysms (AAAs), yet open repair (OR) remains necessary, and the decision hinges on fitness for open surgery. No standardized preoperative tool defines who is “unfit” for OR. We derived and validated a preoperative risk score for severe major adverse events (MAE) after elective OR and examined whether high-risk patients had fewer perioperative events with EVAR. **Methods**: Using the Vascular Quality Initiative (VQI) Open AAA registry, we identified 9833 elective AAA OR patients without prior aortic surgery. Severe MAE was a 30-day composite of death, myocardial infarction (MI), stroke, new dialysis, or bowel ischemia. A multivariable model was converted to an integer score, validated, and benchmarked against the Revised Cardiac Risk Index (RCRI), E-PASS, and a Vascular Quality Initiative Frailty Index. High-risk (score ≥ 7) OR patients were propensity score matched 1:1 to elective EVAR patients and outcomes were compared. **Results**: Severe MAE occurred in 10.8% of OR patients. The 0–18-point score had an optimism-corrected C-statistic of 0.67 and calibration slope of 0.96, and it outperformed RCRI (C 0.57), E-PASS (C 0.61), and a Vascular Quality Initiative Frailty Index (C 0.63) (all ΔAUC *p* < 0.001). Severe MAE rose across low (4%), intermediate (9%), and high (20%) tiers; performance was stable on temporal validation (AUC 0.67, slope 0.94). Among 2357 matched high-risk pairs, severe MAE was 20.3% (OR) vs. 4.4% (EVAR) (risk difference +15.9 percentage points; relative risk 4.6), with the largest absolute benefit in the highest-risk patients. **Conclusions**: A simple preoperative score stratifies perioperative risk for elective open AAA repair and outperforms generic indices. High-risk patients would have had markedly fewer perioperative adverse outcomes if treated with EVAR. These associational findings can inform shared decision-making.

## 1. Introduction

Endovascular aortic repair (EVAR) has become the primary treatment modality for abdominal aortic aneurysms (AAAs) because of its superior perioperative outcomes compared with open repair (OR) [1,2,3,4]. Randomized trials nonetheless show that the early survival advantage of EVAR narrows over time, and open repair remains necessary or preferable for patients with unfavorable anatomy, long life expectancy, or contraindications to endografting [2,4]. The choice between OR and EVAR therefore depends heavily on whether a given patient is “fit” to tolerate an open operation.

Despite the centrality of this judgment, there is no standardized, AAA-specific preoperative tool to define which patients are unfit for open repair. Clinicians rely on gestalt and on generic indices such as the Revised Cardiac Risk Index (RCRI), Estimation of Physiologic Ability and Surgical Stress (E-PASS) score, and Vascular Quality Initiative Frailty Index [5,6,7,8]. A simple, transparent score that estimates the probability of a major adverse event after open AAA repair from routinely available preoperative variables could support more consistent risk communication and operative selection.

We had two objectives. First, we aimed to derive and validate a preoperative integer risk score for a severe major adverse event (MAE)—defined as a 30-day composite of death, myocardial infarction, stroke, new dialysis, or bowel ischemia—after elective open AAA repair using the Vascular Quality Initiative (VQI). Second, we sought to apply the score to identify high-risk patients and examine whether those treated with EVAR experienced fewer perioperative adverse events than comparable patients treated with open repair.

## 2. Materials and Methods

### 2.1. Data Source and Cohort

We analyzed the Society for Vascular Surgery VQI Open AAA and EVAR registries. From the Open AAA file (16,918 records), we included elective procedures, excluding symptomatic and ruptured presentations. We excluded patients who had undergone supramesenteric or unknown clamp positions in the open database. Patients with any prior aortic surgery (open or endovascular) were also excluded. The final derivation cohort comprised 9833 and 45,159 OR and EVAR patients, respectively. Use of the de-identified registry was exempt from individual consent.

### 2.2. Outcome

The primary outcome was a severe MAE, a binary composite of any of the following within 30 days or the index hospitalization: perioperative death (in-hospital death or death within 30 days), myocardial infarction, stroke, new dialysis (temporary or permanent), or bowel/intestinal ischemia.

### 2.3. Missing Data

Missingness among candidate predictors was low for most variables (preoperative creatinine 1.1%, height and weight 0.7%, and each comorbidity indicator < 0.5%) and complete for proximal clamp position, but higher for American Society of Anesthesiologists (ASA) class (8.8%) and preoperative ambulatory status (18.1%). For the primary analysis, we used single imputation in which missing categorical and binary predictors were assigned to their reference (most common) category—equivalent to the variable median—so that a missing comorbidity, ASA class, or ambulatory-status field was treated as absent or normal; missing continuous inputs (creatinine, height, weight) were resolved to the corresponding reference level of the derived variable (for example, a missing body-mass index was classified as neither underweight nor obese, and a missing creatinine as an estimated glomerular filtration rate ≥ 60 mL/min/1.73 m^2^ in non-dialysis patients). Because the score is an additive integer of categorical terms, this is equivalent to conservatively withholding points when information is unavailable. To confirm that imputation did not drive the findings, the open-versus-EVAR comparison was repeated as a complete-case analysis restricted to patients with no missing model covariates, which reproduced the primary result.

### 2.4. Predictors and Score Derivation

Candidate predictors were preoperative and planned-anatomic variables. Estimated glomerular filtration rate (eGFR) was computed from preoperative creatinine using the 2021 race-free CKD-EPI equation [9], anemia used the World Health Organization sex-specific definition (hemoglobin < 13 g/dL in men, <12 g/dL in women) [10], and other severity-graded fields were collapsed to clinically meaningful categories. Comorbidities were entered as binary indicators; severity-graded categorizations of congestive heart failure and COPD were evaluated but did not improve discrimination (C-statistic unchanged) and were not retained, favoring a parsimonious model. A multivariable logistic regression model was fit, retaining covariates by backward elimination (*p* < 0.05). Age and renal function were forced into the model a priori rather than being subject to elimination for two reasons: both are well-established, biologically plausible determinants of perioperative outcome after major vascular surgery, so removing them on the basis of a single cohort’s *p*-value would yield a statistically parsimonious but clinically incoherent model; and both were modeled as ordered categories, forcing the variable to preserve the full dose-response gradient and avoid dropping an individual intermediate category, which would distort the relationship. Regression coefficients were converted to an additive integer point score using the Sullivan (Framingham) method [11], and total scores were grouped into three clinical tiers.

Each predictor’s points were obtained by dividing its regression coefficient by a common anchor (B), set to the mean of the coefficients of the reference one-point comorbidities (hypertension, coronary artery disease, and congestive heart failure), and rounding to the nearest integer, so that one point approximates the effect of a single typical comorbidity; points were then summed to a total (0–18). This scaling preserved predictive performance: the integer score retained essentially the full discrimination of the continuous model (C-statistic 0.672 versus 0.676).

### 2.5. Validation and Benchmarking

Discrimination (C-statistic) and the calibration slope were internally validated using 400 bootstrap resamples with optimism correction; calibration was additionally assessed by the Hosmer–Lemeshow statistic and an observed-versus-predicted plot [12,13]. Temporal robustness was tested by refitting the model in earlier years and evaluating it in contemporary years (and vice versa) [14]. We performed decision-curve analysis [15], evaluated subgroup discrimination, and benchmarked the score against the Revised Cardiac Risk Index (RCRI), E-PASS, and VQI Frailty Index for severe MAE (computing the difference in AUC and the integrated discrimination improvement [IDI] and continuous net reclassification improvement [NRI]) [16]. As an external anchor to clinical judgment, the score was compared with the surgeon-recorded “unfit for open AAA” designation available in the EVAR registry (agreement by kappa).

The frozen score (identical integer weights, without any re-estimation) was externally validated in the American College of Surgeons National Surgical Quality Improvement Program (ACS-NSQIP) Procedure-Targeted open-AAA Participant Use Files (PUF), 2014–2024, each linked to the general Adult PUF by case identifier. We applied the same inclusion criteria (elective, non-supraceliac, open infrarenal/suprarenal repair) and the same severe-MAE definition. We also excluded patients that had prior aortic surgery (Figure S1). It should be noted that by including elective patients with aortic diameter as an indication for treatment, this inherently excluded patients with prior aortic surgery in the NSQIP database. Predictors were mapped to NSQIP fields (eGFR from creatinine, ASA class, functional health status for ambulation, treated hypertension, congestive heart failure, COPD, dialysis, body mass index, and proximal clamp level; bowel ischemia from the procedure-targeted colitis field); coronary artery disease is not recorded in the contemporary NSQIP file and was therefore set to absent, which biases scores conservatively. We assessed discrimination (C-statistic with 2000-sample bootstrap confidence interval), calibration (calibration-in-the-large, calibration slope, and an observed-versus-predicted plot), and observed-versus-expected severe MAE by tier and compared the score with NSQIP’s own estimated probabilities of morbidity and mortality.

### 2.6. High-Risk or Versus EVAR

To examine treatment in high-risk patients, OR patients in the high tier (score ≥ 7) were matched 1:1 without replacement to elective EVAR patients by nearest-neighbor matching on the logit of a propensity score (caliper 0.2 SD), estimated from 13 baseline covariates available in both registries (age, sex, eGFR, dialysis dependence, hypertension, coronary artery disease, congestive heart failure, COPD, diabetes, current smoking, anemia, body mass index, and aneurysm diameter). Balance was assessed by standardized mean differences (SMD; threshold 0.10). Outcomes were severe MAE and its components, as well as all-cause mortality (Kaplan–Meier, log-rank). Inverse-probability-of-treatment weighting (IPTW) and complete-case analysis were performed as sensitivity analyses. Analyses were conducted in Python 3.10.12 (NumPy, NumPy 2.2.6, pandas 2.3.3; figures in Matplotlib 3.10.9); two-sided *p* < 0.05 was significant.

## 3. Results

### 3.1. Cohort and Outcome

Among 9833 elective open AAA repairs (median age 69 years; 26.7% women), severe MAE occurred in 1065 patients (10.8%); the patient-selection flow for both cohorts is shown in Figure S1. The most frequent components were myocardial infarction (465; 4.7%), perioperative death (331; 3.4%), bowel ischemia (329; 3.3%), new dialysis (262; 2.7%), and stroke (70; 0.7%). Baseline characteristics are shown in Table 1.

### 3.2. Risk Score Derivation and Performance

Sixteen factors were retained in the multivariable model (Table 2); the strongest predictors were renal dysfunction (dialysis dependence and eGFR < 30) and advancing age, followed by congestive heart failure, COPD, coronary artery disease, ASA class IV–V, suprarenal clamp, hypertension, impaired ambulation, underweight (body mass index < 18.5 kg/m^2^), and obese class II–III (≥35 kg/m^2^). The additive integer score was created based on the multivariable model and a score could range from 0–18 (Table 3). Discrimination was modest but typical for a composite surgical-morbidity endpoint: optimism-corrected C-statistic 0.672, with negligible optimism, an apparent calibration slope of 1.00 and an optimism-corrected slope of 0.96 (95% bootstrap range 0.88–1.06), and a non-significant Hosmer–Lemeshow test (χ^2^ = 4.3). Observed severe-MAE increased monotonically with the score (Table S1) and across the three risk tiers (Table 4): low (4.0%), intermediate (9.3%), and high (20.3%).

Internal calibration was excellent: calibration-in-the-large was exact (observed = predicted, 10.8%), the apparent calibration slope was 1.00 (optimism-corrected 0.96), and observed severe MAE aligned with predicted risk across deciles (Figure S2). In a sensitivity analysis using center identifiers (242 centers; median 3.5 open AAA repairs per center per year), discrimination was preserved across hospital-volume strata (C-statistic 0.66–0.72), the score remained independently predictive after adjustment for log center annual volume (odds ratio per point 1.34, 95% CI 1.30–1.38; volume itself was not significant, odds ratio 0.96, 95% CI 0.90–1.02), and a center-clustered bootstrap widened the discrimination interval only modestly (0.65–0.69).

### 3.3. Benchmarking, Temporal Validation, and Clinical Utility

The open AAA risk stratification score significantly outperformed every established preoperative tool reproducible in VQI (Table 5). The open AAA risk stratification score also outperformed every established preoperative tool in predicting each component of severe MAE (Table 5). The open AAA risk stratification score predicted perioperative death (C 0.73) and need for dialysis (C 0.72) well; discrimination was weaker for the more technical components (MI, stroke, bowel ischemia; AUC ~0.64–0.66) (Table 5). On out-of-time validation (model trained on 2016 and earlier, tested on 2017–later), discrimination and calibration were preserved (AUC 0.67, calibration slope 0.94; Table S2).

Decision-curve analysis demonstrated greater net benefit than RCRI, E-PASS, the VQI Frailty Index, treat-all, or treat-none strategies across threshold probabilities of approximately 10–50% (Figure S3). Discrimination was broadly stable across age strata, clamp level, and comorbidity burden (AUC 0.63–0.67) (Table S3).

Compared with the surgeon-recorded “unfit for open” designation in the EVAR registry (*n* = 44,677), the continuous score discriminated the clinical flag with an AUC of 0.64, and the proportion designated unfit rose across model tiers (7.0%, 14.1%, 24.4%; Table S4); however, categorical agreement was poor (κ = 0.13), indicating that the model and the operating surgeon identify overlapping but substantially different patients.

### 3.4. High-Risk Patients: Open Versus EVAR

Of the 2358 high-tier (score ≥ 7) open patients, 2357 (99.9%) were matched to an EVAR control; the propensity model c-statistic was 0.81, and all covariates were well balanced after matching (mean|SMD|0.018, maximum 0.036). Among matched pairs, severe MAE occurred in 479 open patients (20.3%) versus 104 EVAR patients (4.4%)—a risk difference of +15.9 percentage points (95% CI +14.1 to +17.7; relative risk 4.6). Every component favored EVAR, including death (7.8% vs. 2.0%), MI (8.8% vs. 1.5%), new dialysis (6.2% vs. 0.8%), and bowel ischemia (5.9% vs. 0.9%) (Table 6).

**Figure 1:**
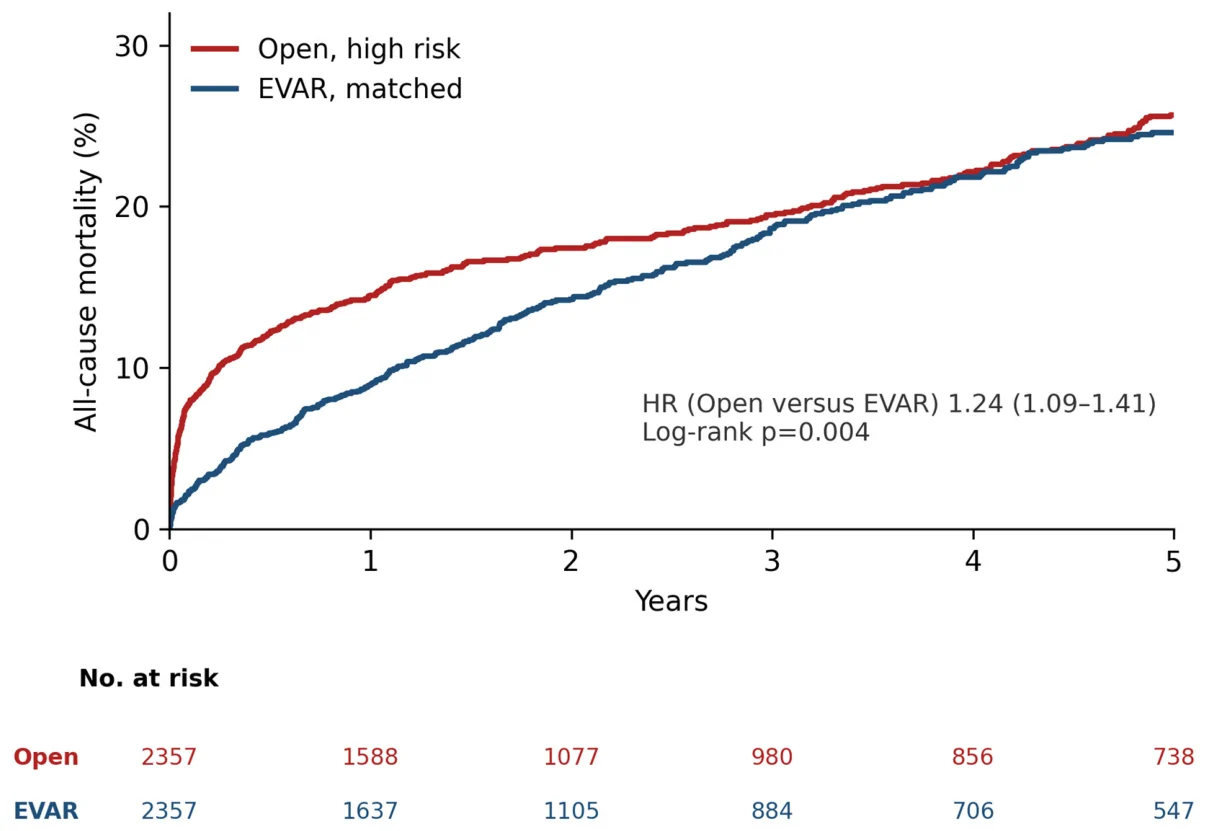
Kaplan–Meier all-cause mortality through 5 years in matched high-risk open versus EVAR patients, with numbers at risk.

Over the course of 5 years, EVAR had a survival advantage amongst high-risk patients (Figure 1). At one year, all-cause mortality remained lower after EVAR (9.0% vs. 14.5%; log-rank *p* < 0.001). However, the survival curves converged thereafter; beyond one year, the difference was no longer statistically significant (landmark log-rank *p* = 0.070) and by 3–5 years, the curves were essentially superimposed (Figure 1). The primary severe-MAE result was unchanged under IPTW (risk difference +16.0 percentage points).

### 3.5. External Validation

**Figure 2:**
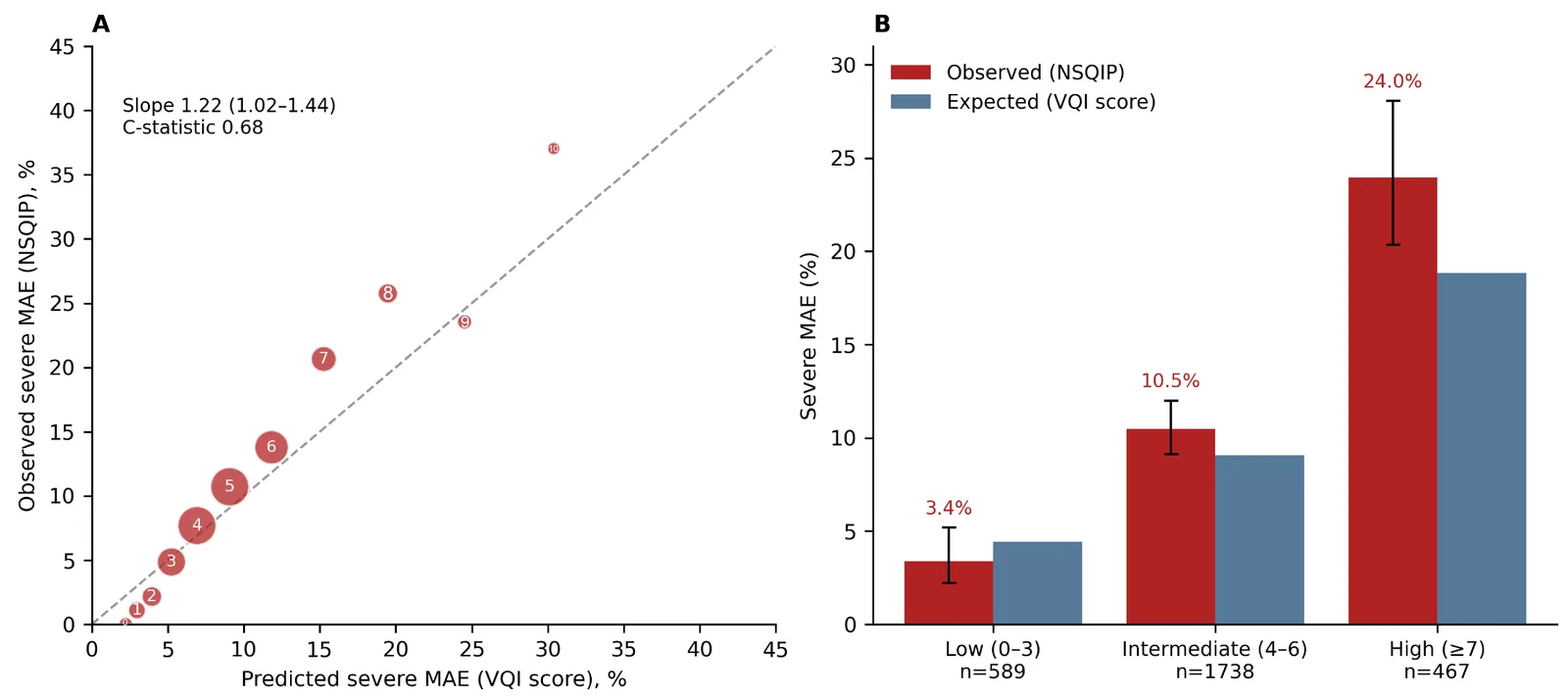
External validation of the severe major adverse event (MAE) risk score utilizing the American College of Surgeons (ACS) National Surgical Quality Improvement Program (NSQIP) database. (**A**) Calibration by score value: each point plots the observed severe MAE rate in NSQIP against the Vascular Quality Initiative (VQI) model-predicted probability for that score, with marker size proportional to the number of patients and the dashed line indicating perfect calibration. Only score values with ≥15 patients are shown. (**B**) Observed versus VQI-expected severe MAE across tiers. Red bars show observed rates with 95% confidence intervals; blue bars show the mean VQI-predicted (expected) rate.

In 2794 independent elective open AAA repairs from ACS-NSQIP (2014–2024), severe MAE occurred in 11.2%, closely matching the derivation cohort (10.8%). The frozen score discriminated severe MAE with a C-statistic of 0.68 (95% CI 0.66–0.71)—equal to the internal estimate—and perioperative mortality with a C-statistic of 0.72 (0.67–0.77). Observed severe MAE rose monotonically across tiers (low 3.4%, intermediate 10.5%, high 24.0%), and the risk gradient and rank-ordering were preserved (calibration slope 1.22, 95% CI 1.02–1.44); however, the frozen score modestly under-predicted absolute risk (observed/expected 1.15), most evident in the high-risk tier (observed 24.0% vs. expected 18.8%; intermediate 10.5% vs. 9.1%; low 3.4% vs. 4.4%). This under-prediction is expected because coronary artery disease could not be mapped in NSQIP and was set to absent. In addition, NSQIP applies stricter definitions for several inputs (i.e., congestive heart failure within 30 days, severe COPD). This in essence deflates scores, particularly among higher-risk patients. Nonetheless, the score out-discriminated NSQIP’s own predicted-morbidity probability for severe MAE (0.68 versus 0.65) and matched its predicted-mortality probability (0.72 versus 0.73) (Figure 2; Table S5).

## 4. Discussion

In a large national registry, we derived and validated a simple preoperative score that stratifies the risk of a severe major adverse event after elective open AAA repair, and we used it to show that patients identified as high-risk (“unfit”) may have experienced markedly fewer perioperative adverse events if treated with EVAR. The score uses routinely available variables, outperforms other well-established risk stratification scores, remained stable on temporal validation, and was externally validated.

Several findings merit emphasis. First, the discrimination for the composite endpoint was modest (C = 0.67), which is characteristic of aggregated surgical-morbidity outcomes. However, the risk score derived in this study discriminated death (C = 0.73) and renal failure (C = 0.72) far better than the more idiosyncratic components (MI, stroke, bowel ischemia). The composite is therefore best understood as a broad morbidity-and-mortality stratifier, with the option of a mortality-focused application when a sharper endpoint is desired. This is important given that the study raises an important conceptual consideration regarding the definition of operative risk: whether clinicians aim primarily to avoid short-term mortality or to minimize the occurrence of the more severe major adverse events. We contend that the latter is a more meaningful goal, as MAEs are strongly associated with diminished long-term survival and quality of life [17,18,19,20,21]. Nonetheless, if someone had the perspective that only mortality should be considered, the risk tool created in this study performs well for predicting mortality.

We also found that the scoring system derived from this study outperformed every established preoperative risk score that could be reproduced in this registry—RCRI (ΔAUC + 0.10; NRI 0.40), the E-PASS preoperative risk score (ΔAUC + 0.06), and a Vascular Quality Initiative Frailty Index (ΔAUC + 0.04), all *p* < 0.001. These differences are expected because RCRI captures only cardiac risk, E-PASS is a generic physiologic-ability index, and the frailty index is an equally weighted comorbidity count, whereas the dominant drivers of severe MAE after open AAA repair are renal dysfunction and age. Currently, the Society for Vascular Surgery (SVS) has the RCRI prediction tool available on their website to guide physicians’ decision-making between OR and EVAR. We argue that isolating clinical judgement on cardiac risk alone is not appropriate for the reasons listed above. Bryce et al. similarly found that current risk stratification scores did not perform well predicting all-cause mortality, major adverse cardiac events, and cardiac death [5]. Further, the complexity of the scoring systems made it difficult to use it clinically [5]. The scoring system derived in this study outperformed current scoring systems and is much simpler to use in a clinical setting.

In addition, it does not appear that physicians are able to correctly identify patients who are high-risk for open repair, emphasizing the need for a clinically applicable risk stratification tool. The VQI EVAR database captures data on whether a surgeon is performing an endovascular repair on a patient because they deemed them unfit. We found that our risk prediction scoring system correlated with but did not replicate the operating surgeon’s “unfit” judgment (κ = 0.13). The model flagged a sizeable high-risk group the surgeon did not and vice versa, suggesting that an explicit, reproducible score carries information complementary to clinical gestalt and could reduce variability in operative selection.

Among comparable high-risk patients, EVAR was associated with a roughly four- to five-fold lower rate of perioperative severe MAE and lower 1-year mortality. More interestingly, the survival benefit of EVAR in high-risk patients diluted after one year, which mirrors the mortality data in EVAR-1, DREAM, and OVER [1,2,3]. The loss in survival benefits following EVAR is thought to be due to the lack of durability and high reintervention rates. Unfortunately, the VQI database was not robust enough to analyze the long-term reintervention burden. These results should therefore inform, but not dictate, treatment selection, and must be weighed against the durability and life-expectancy trade-offs of OR versus EVAR demonstrated in prior studies; a patient at high risk for open repair is not automatically an optimal candidate for EVAR, whose suitability depends on anatomy, frailty, life expectancy, and expected durability. Further, this also needs to be balanced with rupture risk, especially in high-risk patients. The aortic-related mortality in high-risk patients should be considered a significant proportion of their predicted all-cause mortality, and one can make an argument of raising the threshold for repair for high-risk groups [22,23].

This study has important limitations. It is observational; the OR-versus-EVAR comparison is associational and not a causal or randomized treatment-effect estimate, and residual confounding by anatomy and frailty is likely despite matching. VQI complications are recorded for the index hospitalization only, so post-discharge readmissions within 30 days are incompletely ascertained. Detailed anatomic variables were not modeled and likely contribute to the performance ceiling. Long-term follow-up in VQI was too sparse to assess reintervention or durability (records reached 2 years in <5% of patients). Despite these limitations, the VQI was selected due to its large size, longitudinal structure, and comprehensive capture of infrarenal abdominal aortic aneurysm cases. However, because the model was derived from VQI-participating centers, its generalizability to non-participating institutions may be limited. Validation was internal and temporal only; external validation in an independent cohort is required before the score is used as a clinical decision tool, and claims of clinical applicability are tempered accordingly.

Two caveats temper clinical application. First, discrimination for the composite endpoint was moderate (C-statistic ≈ 0.68), as expected for an aggregated surgical-morbidity outcome whose components are partly procedure-driven; the score performed better for mortality alone (C-statistic 0.73–0.74) and is best used to make risk communication consistent and to flag the extremes of the risk distribution, complementing rather than replacing surgical and multidisciplinary judgment. Second, neither registry captures detailed aneurysm morphology—neck length and angulation, iliac anatomy, thrombus burden, and calcification—which strongly influences operative complexity, clamp position, operative time, and blood loss and likely contributes to the performance ceiling; the score therefore reflects preoperative physiology rather than anatomic operability. Frailty is likewise multidimensional, and only functional status and body-mass-index extremes were available; structured frailty instruments (for example the Clinical Frailty Scale), sarcopenia, and nutritional and cognitive measures are promising additions for future iterations.

Future directions include machine-learning and artificial-intelligence models that incorporate anatomic and frailty data, dynamic intra- and postoperative risk updating, integration into the electronic health record for point-of-care use, and prospective multicenter validation.

## 5. Conclusions

In a large national registry, a simple preoperative integer score stratified the risk of a severe perioperative adverse event after elective open AAA repair, outperformed established preoperative risk scores (RCRI, E-PASS, and a VQI frailty index) and NSQIP’s own risk probabilities, and—importantly—retained its discrimination and risk gradient on fully external validation in ACS-NSQIP. The patients the score identifies as physiologically high-risk—driven chiefly by renal dysfunction, advancing age, cardiopulmonary comorbidity, poor functional status, and body-mass-index extremes—are those in whom the hazard of open repair is greatest and in whom endovascular repair was associated with markedly fewer perioperative adverse events and lower early mortality. These associations are observational and should inform, not dictate, treatment. A high-risk designation for open repair does not by itself make a patient an ideal candidate for EVAR. Selection must integrate aneurysm anatomy, life expectancy, frailty, expected durability and reintervention burden, and patient preference within multidisciplinary, shared decision-making. Positioned this way—as a transparent, reproducible complement to surgical judgment rather than a substitute for it—the score can make preoperative risk communication and operative selection more consistent. Prospective, multicenter validation and the integration of anatomic and frailty data are the natural next steps.

## Figures and Tables

**Table 1:** Baseline characteristics of the derivation cohort (N = 9833).

Characteristic	Value
Age, years—median (IQR)	69 (64–75)
Female sex	2624 (26.7%)
Current smoker	4273 (43.5%)
Hypertension	8211 (83.5%)
Diabetes mellitus	1614 (16.4%)
Coronary artery disease	2537 (25.8%)
Congestive heart failure	709 (7.2%)
Chronic obstructive pulmonary disease	3319 (33.8%)
Dialysis-dependent	48 (0.5%)
eGFR < 60 mL/min/1.73 m^2^	2446 (24.9%)
ASA class IV–V	2837 (28.9%)
Anemia (Hgb < 13 M/<12 F)	2153 (21.9%)
Impaired ambulation	503 (5.1%)
Underweight (BMI < 18.5)	297 (3.0%)
Obese class II–III (BMI ≥ 35)	789 (8.0%)
Max AAA diameter, mm—median (IQR)	57 (53–63)
Proximal clamp position	—
Infrarenal	5977 (60.8%)
Above one renal	1529 (15.5%)
Above both renals	2327 (23.7%)

IQR, interquartile range; eGFR, estimated glomerular filtration rate; ASA; American Society of Anesthesiologists Physical Status Classification; Hgb, hemoglobin; BMI, body mass index; AAA, abdominal aortic aneurysm; M, male; F, female.

**Table 2:** Multivariable logistic regression for severe MAE (open AAA cohort).

Predictor (Adjusted)	OR	95% CI	*p*
**Demographics**			
Age 60–69 (vs. <60)	1.66	1.24–2.24	<0.001
Age 70–79 (vs. <60)	2.47	1.85–3.31	<0.001
Age ≥ 80 (vs. <60)	3.36	2.42–4.68	<0.001
**Renal function**			
eGFR 45–59 (vs. ≥60)	1.19	0.99–1.42	0.059
eGFR 30–44	1.91	1.56–2.34	<0.001
eGFR < 30 (non-dialysis)	3.46	2.55–4.69	<0.001
Dialysis-dependent	3.69	1.89–7.17	<0.001
**Cardiovascular and other comorbidity**			
Hypertension	1.30	1.06–1.60	0.013
Coronary artery disease	1.29	1.12–1.49	<0.001
Congestive heart failure	1.40	1.13–1.73	0.002
Chronic obstructive pulmonary disease	1.39	1.22–1.59	<0.001
**Operative**			
ASA class 4–5	1.27	1.11–1.47	<0.001
Suprarenal clamp—above one renal	1.21	1.01–1.45	0.041
Suprarenal clamp—above both renals	1.33	1.15–1.55	<0.001
**Functional and nutritional**			
Impaired ambulation	1.32	1.03–1.70	0.029
Underweight (BMI < 18.5)	1.66	1.22–2.27	0.001
Obese class II–III (BMI ≥ 35)	1.34	1.06–1.69	0.014

OR, odds ratio; CI, confidence interval; eGFR, estimated glomerular filtration rate; ASA, American Society of Anesthesiologists Physical Status Classification; BMI, body mass index.

**Table 3:** Integer point score.

Risk Factor	Points
Age 60–69 y	+2
Age 70–79 y	+3
Age ≥ 80 y	+4
eGFR 45–59	+1
eGFR 30–44	+2
eGFR < 30 (non-dialysis)	+4
Dialysis-dependent	+5
Hypertension	+1
Coronary artery disease	+1
Congestive heart failure	+1
Chronic obstructive pulmonary disease	+1
ASA class IV–V	+1
Proximal clamp above one or both renals	+1
Impaired ambulation	+1
Underweight (BMI < 18.5)	+2
Obese class II–III (BMI ≥ 35)	+1

eGFR, estimated glomerular filtration rate; ASA, American Society of Anesthesiologists Physical Status Classification; BMI, body mass index.

**Table 4:** Risk tiers and observed severe MAE.

Tier	Score	n	Observed Severe MAE
Low	0–3	2076	4.0% (3.2–4.9)
Intermediate	4–6	5399	9.3% (8.6–10.1)
High	≥7	2358	20.3% (18.7–22.0)

**Table 5:** Discrimination (AUC, 95% CI) of the risk score versus established preoperative scores for the composite severe-MAE endpoint and each component (open AAA cohort, n = 9833).

Outcome (Events)	Present Score	RCRI	E-PASS	VQI-FS
Severe MAE (1065)	0.67 (0.66–0.69)	0.57 (0.56–0.59)	0.61 (0.59–0.63)	0.63 (0.61–0.65)
Death (331)	0.73 (0.71–0.76)	0.61 (0.59–0.64)	0.65 (0.62–0.68)	0.68 (0.65–0.71)
Myocardial infarction (465)	0.66 (0.63–0.68)	0.58 (0.55–0.60)	0.62 (0.59–0.64)	0.62 (0.60–0.65)
Stroke (70)	0.65 (0.59–0.71)	0.59 (0.53–0.66)	0.57 (0.49–0.63)	0.65 (0.59–0.71)
New dialysis (262)	0.72 (0.69–0.75)	0.62 (0.58–0.65)	0.61 (0.58–0.64)	0.68 (0.65–0.71)
Bowel ischemia (329)	0.64 (0.61–0.67)	0.54 (0.51–0.57)	0.59 (0.56–0.61)	0.60 (0.57–0.63)

**Table 6:** Matched outcomes in high-risk (unfit) patients: open vs. EVAR (2357 pairs/arm).

Outcome	Open n (%)	EVAR n (%)	Risk Diff (95% CI)	RR
Severe MAE	479 (20.3%)	104 (4.4%)	+15.9 [14.1, 17.7]	4.6
Death	184 (7.8%)	46 (2.0%)	+5.9 [4.6, 7.1]	4.0
Myocardial infarction	207 (8.8%)	36 (1.5%)	+7.3 [6.0, 8.5]	5.8
Stroke	29 (1.2%)	6 (0.3%)	+1.0 [0.5, 1.5]	4.8
New dialysis	147 (6.2%)	19 (0.8%)	+5.4 [4.4, 6.5]	7.7
Bowel ischemia	140 (5.9%)	25 (1.1%)	+4.9 [3.8, 5.9]	5.6

## Data Availability

The data used for this study was from the Society for Vascular Surgery Vascular Quality Initiative.

## Supplementary Materials

The following supporting information can be downloaded at https://www.mdpi.com/article/10.3390/jcm15166206/s1, Table S1: Observed severe major adverse event (MAE) rate by total risk-score value, open AAA cohort (n = 9833); Table S2: Temporal (out-of-time) validation of the risk model; Table S3: Discrimination of the risk score within prespecified subgroups; Table S4: Agreement between the risk score and the surgeon-recorded “unfit for open repair” designation, EVAR registry (n = 44,677); Table S5: Variable mapping from the Vascular Quality Initiative (derivation) to ACS-NSQIP (external validation); Figure S1: Study flow-chart; Figure S2: Internal calibration of the severe major adverse events (MAEs) risk score in the Vascular Quality Initiative (VQI) derivation cohort. Observed versus model-predicted probability of a severe MAE, grouped by tenths (deciles) of predicted risk; Figure S3: Decision-curve analysis of the severe major adverse event risk score, Vascular Quality Initiative Frailty Index (VQI-FI), Estimating the Physiologic Ability and Surgical Stress (E-PASS) score, Revised Cardiac Risk Index (RCRI), treat all strategy, and the treat none strategy.

## Abbreviations

EVAR	endovascular aortic repair
OR	open repair
