# Supplementary material for: Risk Stratification of Patients Being Considered for Open Abdominal Aortic Aneurysm Repair
Source: J Clin Med. 2026 Aug 11;15(16):6206. doi: 10.3390/jcm15166206 (PMC13512917; doi:10.3390/jcm15166206)
Supplement: Supplementary file 1 [file jcm-15-06206-s001.zip › jcm-4447907-supplementary.pdf]

Supplementary Material

Table S1. Observed severe major adverse event (MAE) rate by total risk-score value, open AAA cohort (n = 9833).

Score	Risk tier	n	Events	Severe MAE % (95% CI)
0	Low	97	3	3.1 (1.1–8.7)
1	Low	269	8	3.0 (1.5–5.8)
2	Low	554	16	2.9 (1.8–4.6)
3	Low	1,156	56	4.8 (3.7–6.2)
4	Intermediate	1,713	126	7.4 (6.2–8.7)
5	Intermediate	2,048	188	9.2 (8.0–10.5)
6	Intermediate	1,638	189	11.5 (10.1–13.2)
7	High	1,113	186	16.7 (14.6–19.0)
8	High	610	117	19.2 (16.3–22.5)
9	High	338	77	22.8 (18.6–27.5)
10	High	164	44	26.8 (20.6–34.1)
11	High	91	36	39.6 (30.1–49.8)
12	High	34	13	38.2 (23.9–55.0)
13	High	7	5	71.4 (35.9–91.8)
14	High	1	1	100.0 (20.7–100.0)

Table S2. Temporal (out-of-time) validation of the risk model.

Validation	AUC	Calibration slope
Trained ≤2016 → tested 2017–2022	0.67	0.94
Trained 2017–2022 → tested ≤2016	0.66	0.94

Table S3. Discrimination of the risk score within prespecified subgroups.

Subgroup	n	Severe MAE	AUC
Age <70	4,967	7.6%	0.66
Age >=70	4,866	14.1%	0.63
Infrarenal clamp	5,977	9.5%	0.67
Suprarenal clamp	3,856	12.9%	0.66
Comorbidity 0-2	8,604	9.7%	0.66
Comorbidity 3+	1,229	18.5%	0.65

Table S4. Agreement between the risk score and the surgeon-recorded "unfit for open repair" designation, EVAR registry (n = 44,677).

Model tier	n	Clinician "unfit"	EVAR severe MAE
Low (0–3)	8,112	6.6%	0.6%
Intermediate (4–6)	28,003	13.7%	1.6%
High (≥7)	8,562	24.1%	4.5%

Table S5. Variable mapping from the Vascular Quality Initiative (derivation) to ACS-NSQIP (external validation).

Score predictor	NSQIP field(s)	Mapping
Age band (60–69/70–79/≥80)	Age	Direct
eGFR ladder (45–59/30–44/<30)	PRCREAT + Age + SEX (CKD-EPI 2021)	Direct
Dialysis-dependent	DIALYSIS	Direct
Hypertension	HYPERMED	Direct
Coronary artery disease	— (not recorded in NSQIP PUF)	Set to absent (conservative)
Congestive heart failure	HXCHF	Direct
COPD	HXCOPD	Direct
ASA class IV–V	ASACLAS (4-Life Threat, 5-Moribund)	Direct
Suprarenal clamp	AAA_PCL (above one renal / between SMA & renals)	Direct (targeted file)
Impaired ambulation	FNSTATUS2 (partially/totally dependent)	Proxy (functional status)
Underweight (<18.5) / Obese II–III (≥35)	HEIGHT, WEIGHT → BMI	Direct
Outcome — death (≤30 d)	DOpertoD ≤30	Direct
Outcome — myocardial infarction	CDMI	Direct
Outcome — stroke	CNSCVA	Direct
Outcome — new dialysis	OPRENAFL (acute renal failure / postop dialysis)	Direct
Outcome — bowel ischemia	AAA_COLITIS (targeted file)	Direct

Applied to the ACS-NSQIP Procedure-Targeted open-AAA files (2014–2024) linked to the general Adult PUF by case identifier. See Figure 5 (external-validation performance) and Figure 3 (internal calibration). Coronary artery disease is not captured in the contemporary NSQIP PUF and was set to absent, biasing the score conservatively (maximum attainable score 13 vs 14).

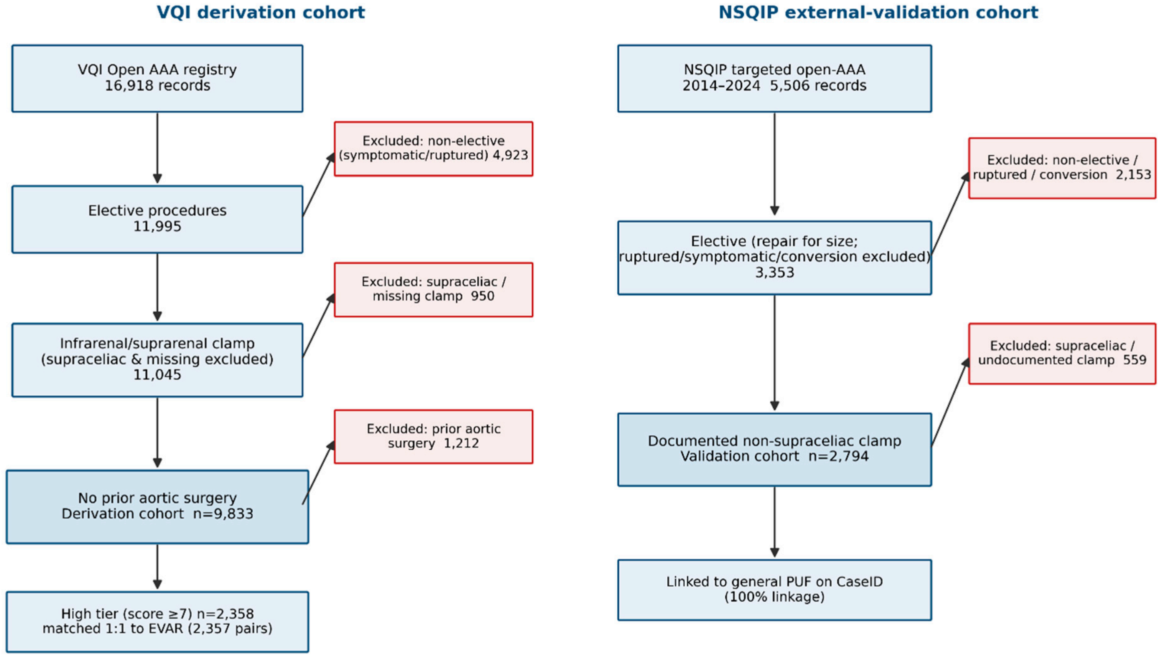

Figure S1. Study flow-chart.

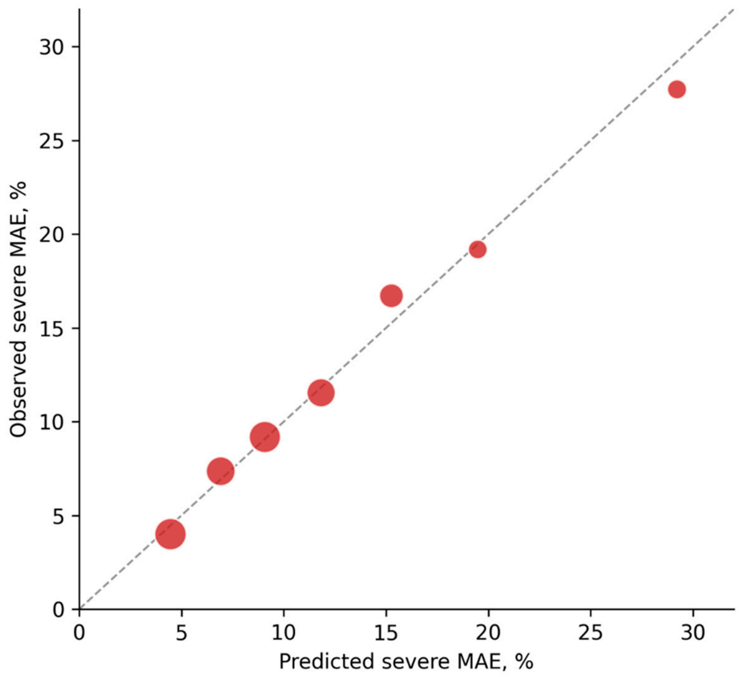

Figure S2. Internal calibration of the severe major adverse events (MAEs) risk score in the Vascular Quality Initiative (VQI) derivation cohort. Observed versus model-predicted probability of a severe MAE, grouped by tenths (deciles) of predicted risk.

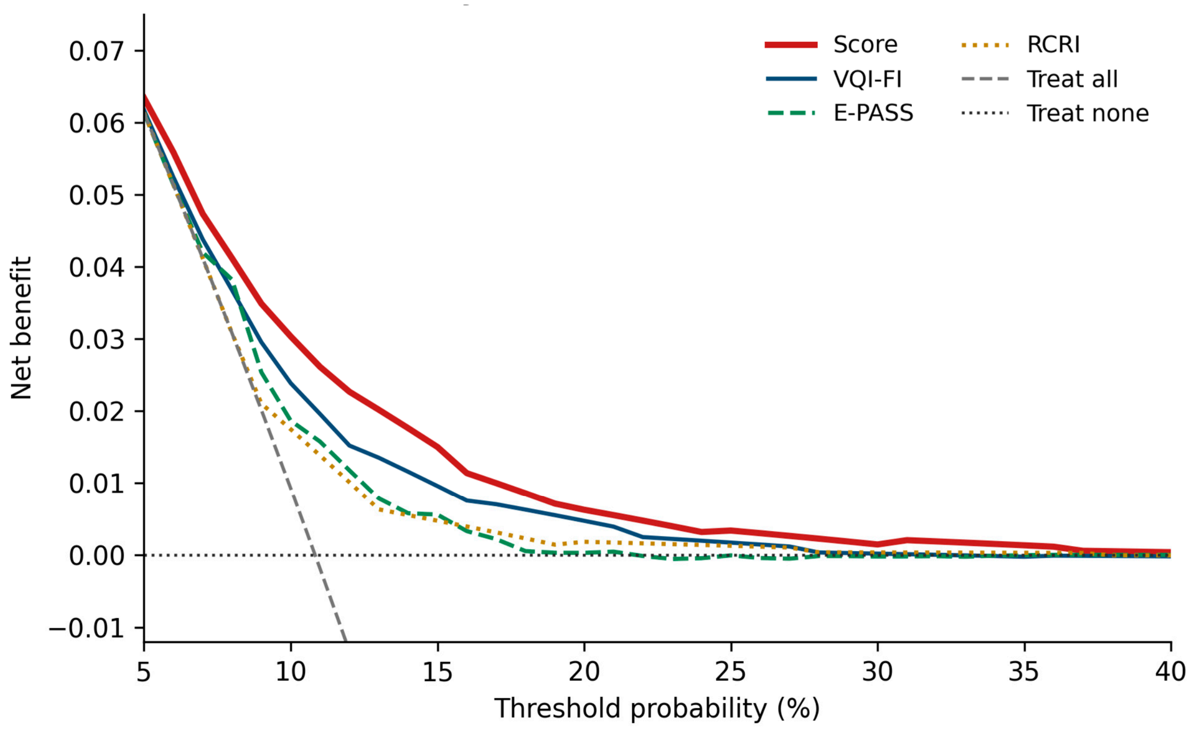

Figure S3. Decision-curve analysis of the severe major adverse event risk score, Vascular Quality Initiative Frailty Index (VQI-FI), Estimating the Physiologic Ability and Surgical Stress (E-PASS) score, Revised Cardiac Risk Index (RCRI), treat all strategy, and the treat none strategy.
